# Anesthetic management of a patient with situs inversus totalis undergoing coronary artery bypass grafting surgery: a case report

**DOI:** 10.1186/s40981-021-00431-1

**Published:** 2021-03-29

**Authors:** Chigusa Nakasone, Masafumi Kanamoto, Wataru Tatsuishi, Tomonobu Abe, Shigeru Saito

**Affiliations:** 1 Department of Anesthesiology and Intensive Care Unit, Gunma University Graduate School of Medicine, Maebashi, Gunma Japan; 2 Department of Cardiovascular Surgery, Gunma University Graduate School of Medicine, Maebashi, Gunma Japan

**Keywords:** Situs inversus totalis, Coronary artery bypass grafting surgery, Anesthetic management

## Abstract

**Background:**

Anesthetic management of coronary artery bypass grafting surgery (CABG) in a dextrocardia patient with situs inversus totalis is rarely encountered and seldom reported in the literature.

**Case presentation:**

A 76-year-old Japanese female patient had been diagnosed with situs inversus totalis and coronary artery disease of 3 vessels, and she subsequently underwent elective CABG. A preoperative examination showed almost normal results. ECG showed right deviation with the normal lead position. In the operating room, ECG leads were applied in reverse. Pulmonary artery catheterization was performed via the left internal jugular vein. A transesophageal echocardiography (TEE) probe was introduced without difficulty. A different angle was needed to acquire the desired views because of her atypical anatomy.

**Conclusion:**

Careful perioperative evaluation, intraoperative management, and inspection of multiplane angle and probe adjustments in TEE are needed for anatomically abnormal patients.

## Introduction

Dextrocardia with situs inversus totalis is a very rare congenital abnormality characterized by the formation of all organs in the mirror image of the normal position, and it has an incidence rate of 1 in 10,000 [1]. The incidence of coronary artery disease in those with this abnormality is the same reported to be normal [2]. Patients with situs inversus totalis are asymptomatic and are usually able to lead a normal life. However, understanding of this anatomical abnormality is important for interpreting patient symptoms accurately and avoiding clinical errors. Furthermore, an accurate understanding of the anatomy in situs inversus totalis can facilitate optimal anesthetic management for these patients, especially when hemodynamic monitoring devices are required, as in cardiac surgery. Herein, we report the case of a 76-year-old female patient with situs inversus totalis who underwent coronary artery bypass grafting surgery (CABG).

## Case report

**Fig. 1:**
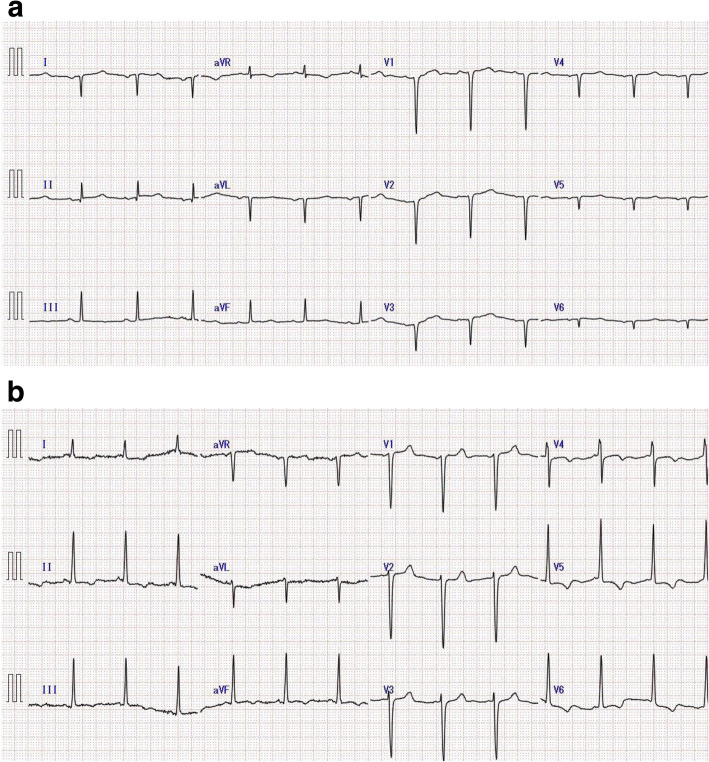
**a** Preoperative electrocardiogram conducted with chest leads in their normal positions. The P wave is negative in the I and aVL leads and positive in the aVR lead. **b** Preoperative ECG conducted with the placement of the right lead reversed showed ST depression in the I and aVL, V5, and V6 leads, and negative T wave in the II, III, aVf, and V4–6 leads

A 76-year-old Japanese female patient had been diagnosed with situs inversus totalis 30 years ago and had since experienced no symptoms of her condition. She had chest pain with physical effort, and coronary angiography was performed. Coronary artery disease of 3 vessels was revealed: the left anterior descending artery (LAD), left circumflex brunch (LCx), and right coronary artery (RCA). She was scheduled to undergo elective CABG. The results of a preoperative chest X-ray were almost within normal limits except her heart was directed to the right. A preoperative electrocardiogram (ECG) was conducted with chest leads in their normal positions; it showed a negative P wave in the I and aVL leads and a positive P wave in the aVR lead, which is a typical right-axis deviation pattern (Fig. 1a). Another ECG was then conducted with the placement of the right lead reversed, which showed ST depression in the I, aVL, V5, and V6 leads, and a negative T wave in the II, III, aVf, and V4-6 leads (Fig. 1b). Preoperative physical examination and evaluation did not reveal any other significant findings.

**Fig. 2:**
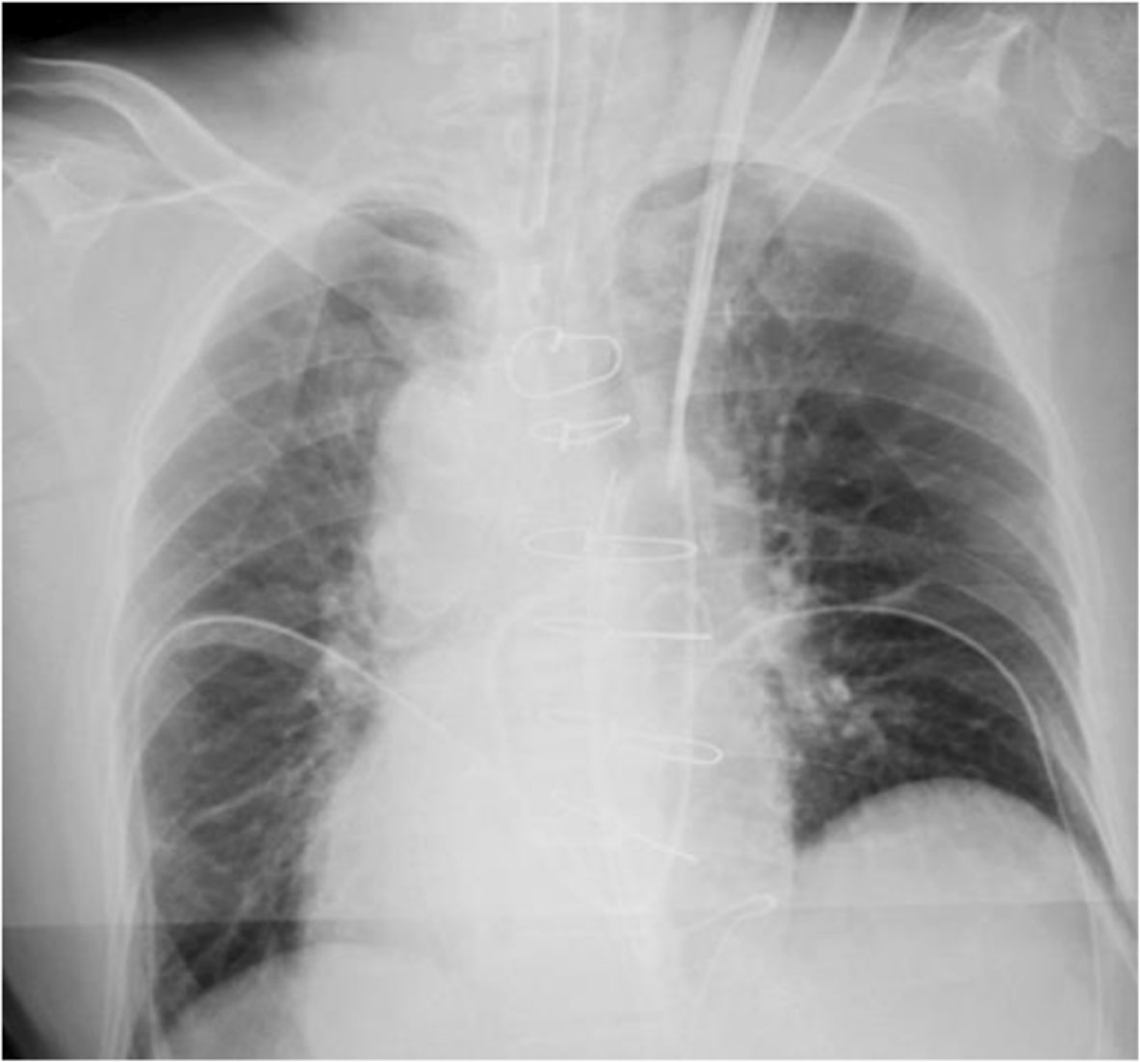
A postoperative chest X-ray revealed that the pulmonary artery catheter was maintained as the mirror image of its normal position

In the operating room, the position in which the ECG leads were applied was reversed. Other standard monitoring methods were applied, including right radial arterial cannulation. Anesthetic induction was performed with propofol/fentanyl, remifentanil, and rocuronium. She was intubated with a tracheal tube, and pulmonary artery catheterization was performed via the left internal jugular vein using a Swan-Gants catheter (Edwards Lifesciences, USA) with the tip placed at the left pulmonary artery. Catheterization was performed successfully and uneventfully. A postoperative chest X-ray revealed that the pulmonary artery catheter was maintained as the mirror image of its normal position (Fig. 2).

**Fig. 3:**
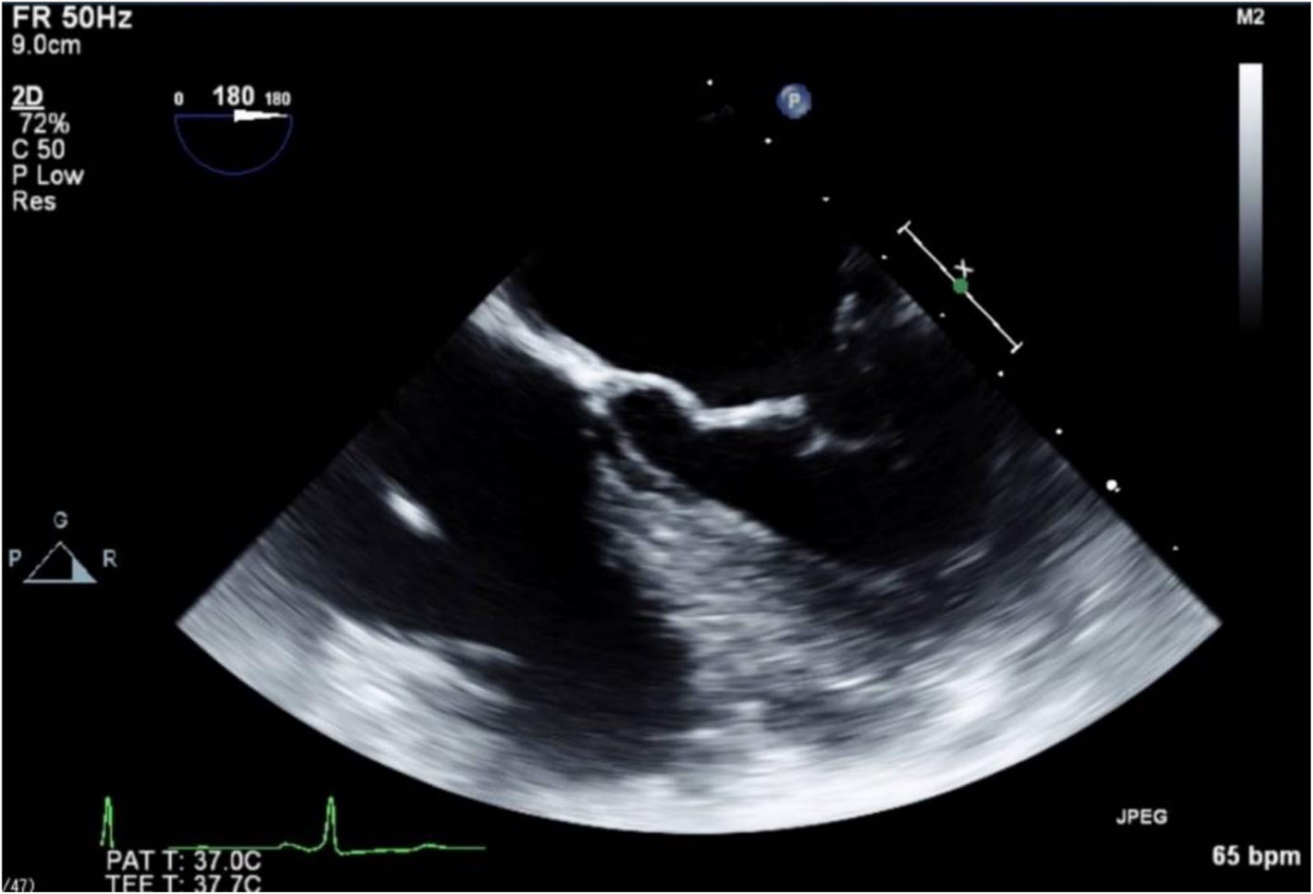
The ME 4-chamber view, which is obtained at a 180°angle

**Fig 4:**
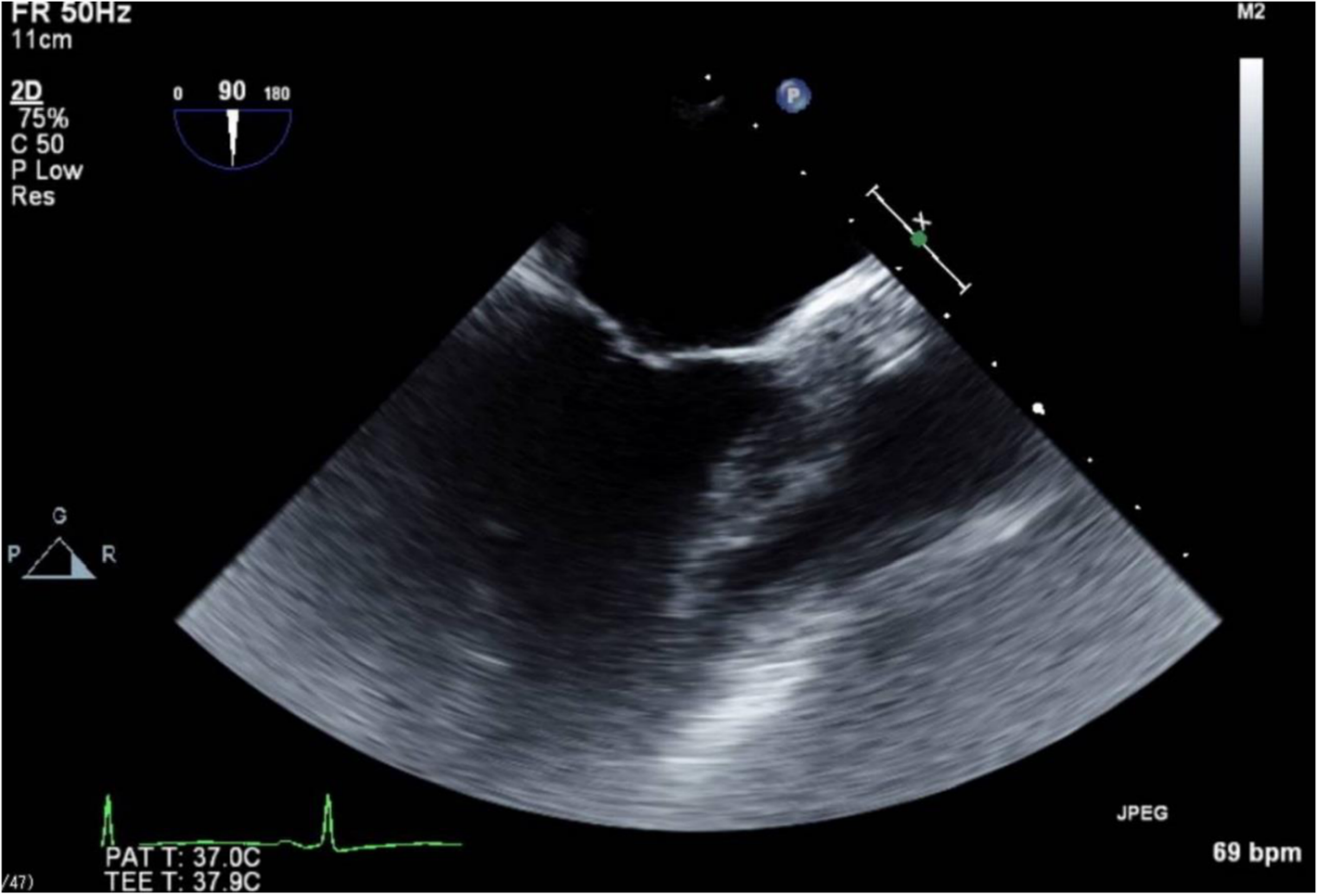
The ME 2-chamber view

A transesophageal echocardiography (TEE) probe was introduced without difficulty, and intraoperative TEE images were recorded. In this patient, a different angle was needed to acquire the desired views because of her atypical anatomy. Midesophageal (ME) 4-chamber and transgastric short-axis views, which are obtained near 0° in normal hearts, were obtained with the left ventricle on the left side of the TEE screen, which is the inverse of the normal TEE view, i.e., inverted 180° (Fig. 3). The ME commissure view, which is normally obtained at 60°, was obtained at 120°. The ME 2-chamber view was obtained at 90° as with the typical anatomy (Fig. 4). The ME long-axis view, normally found at 120°, was obtained at 60°.

**Fig. 5:**
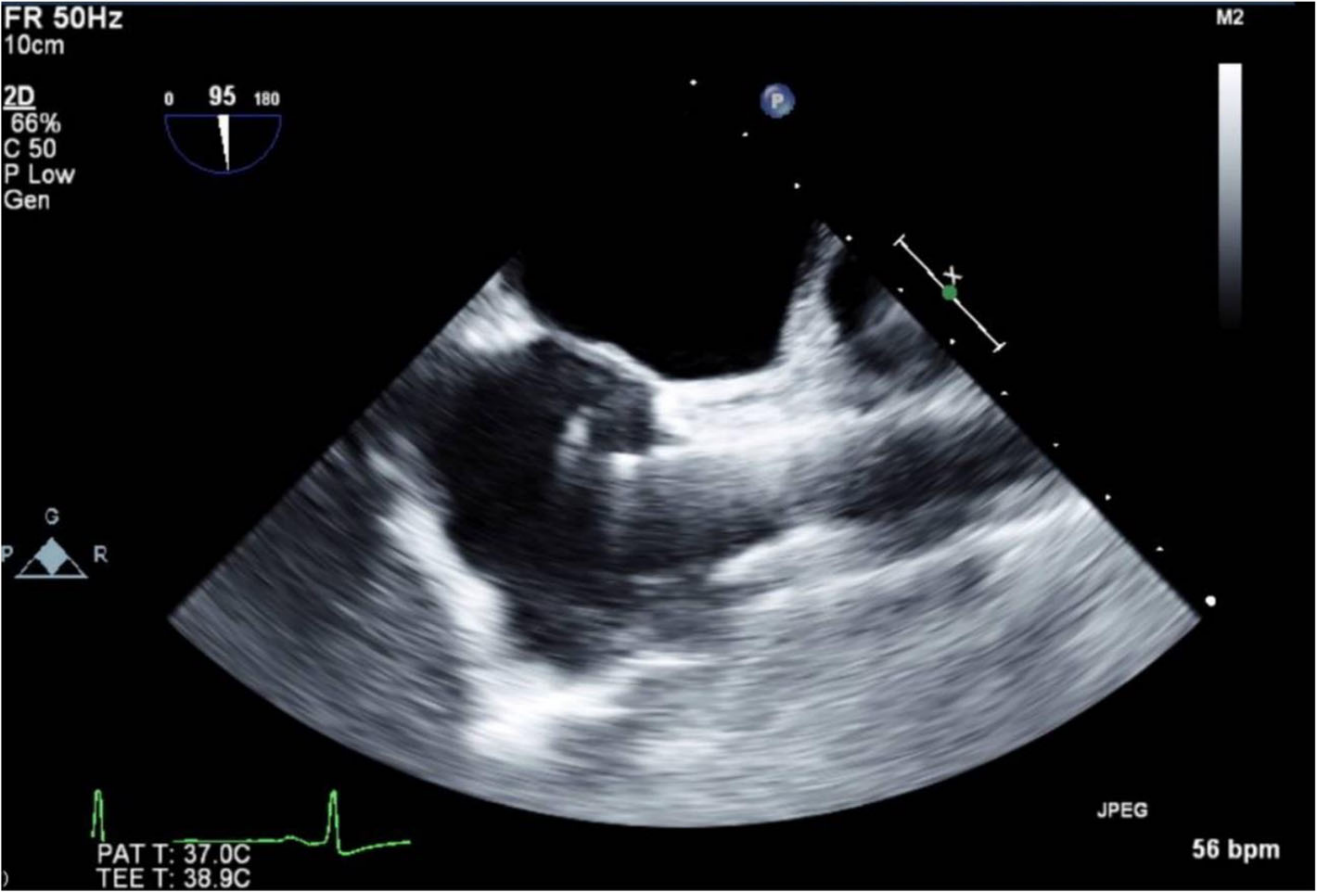
The bicaval view was obtained at 90°

**Fig. 6:**
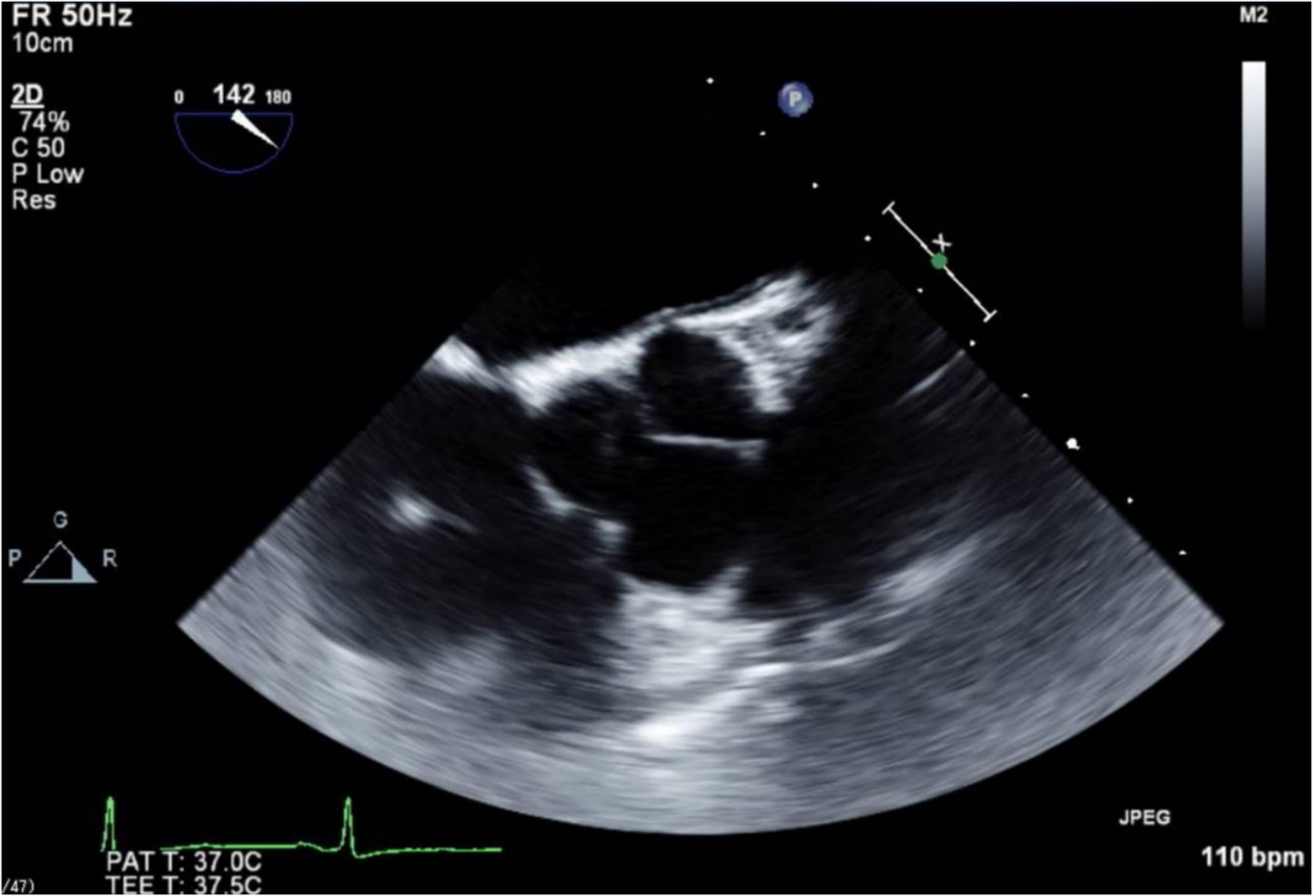
The aortic valve short-axis view, normally found at 30–40°, was obtained at 140–150°

**Fig. 7:**
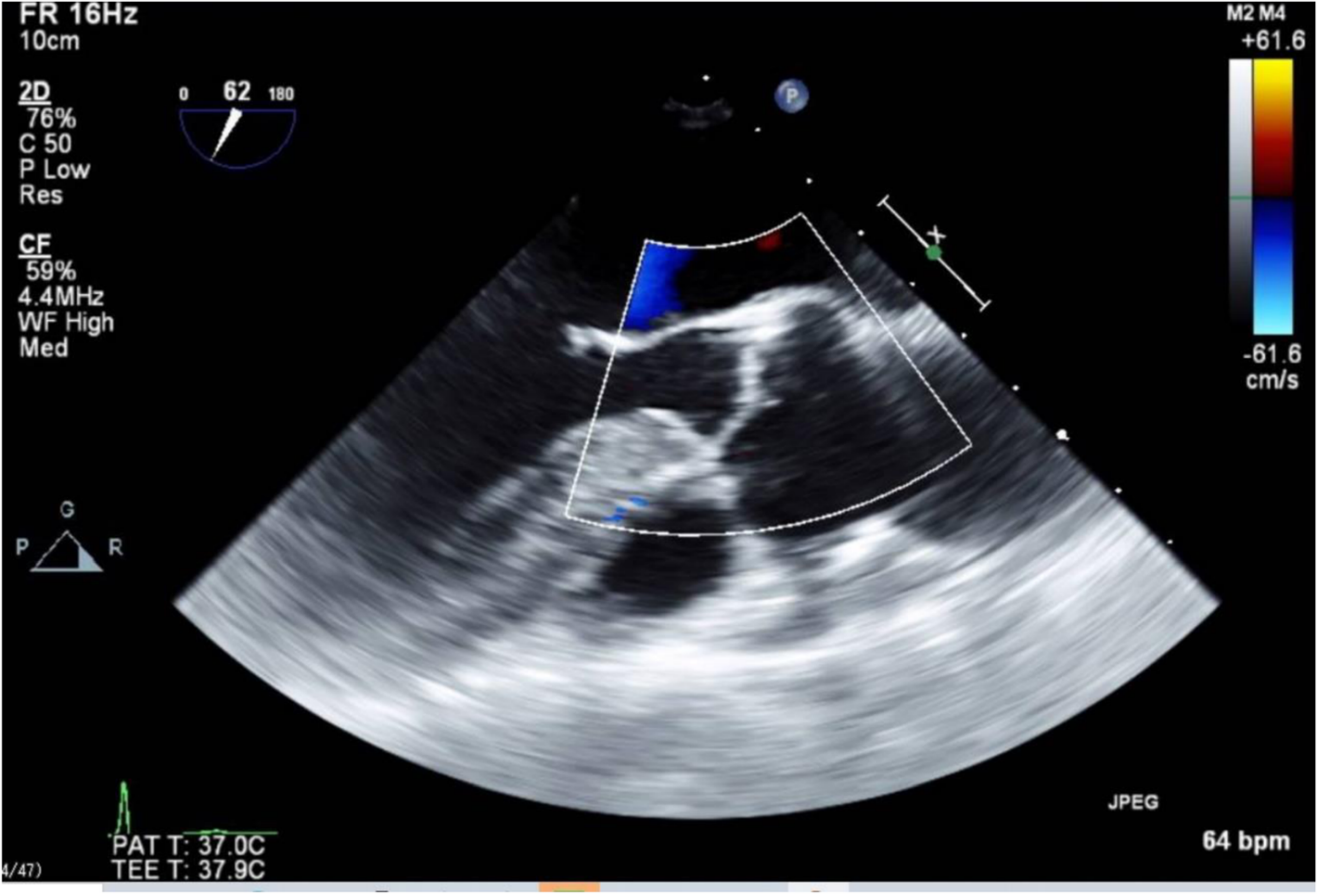
The aortic valve long-axis view, normally found at 120°, was obtained at 60°

The bicaval view was obtained at 90° while rotating the probe to the left in the direction of the right atrium (Fig. 5). The aortic valve short-axis view, normally found at 30–40°, was obtained at 140–150° (Fig. 6). The aortic valve long-axis view, normally found at 120°, was obtained at 60° (Fig. 7). These views were best obtained at a probe angle which differed by 180° from the typical probe angle.

The preparations were completed, and the cardiac surgery was then started. After median sternotomy, the right internal thoracic artery (RITA) and left internal thoracic artery (LITA) were harvested; the RITA was anastomosed to the LAD, the free LITA was anastomosed to the obtuse marginal branch and ascending aorta, and the saphenous venous graft was anastomosed to the RCA. The operating surgeon was on the left side of the patient during the procedure, and the operation was performed without complications. After surgery, the patient was transferred to the intensive care unit, extubated after 4 h of mechanical ventilation, and discharged without complications. The patient was stable during and after the operation. The patient was transferred to the general ward after 4 days in the intensive care unit and discharged on day 17 after the surgery without any complications.

## Discussion

Situs inversus totalis is a rare congenital condition in which the position of all internal organs is the mirror image of their normal anatomies, and it has an incidence rate of 1 in 10,000 [1]. Situs inversus totalis is mainly an autosomal recessive disorder but is sometimes related to X chromosomes. Although most patients with situs inversus totalis have normal life expectancies without any significant related morbidities, they should be meticulously evaluated because the condition may coexist with other congenital comorbidities in the cardiovascular, respiratory, and digestive systems [3]. When situs inversus totalis is combined with recurrent respiratory infections such as sinusitis and bronchiectasis in a patient, it is diagnosed as Kartagener’s syndrome or primary ciliary dyskinesia. Repeated respiratory infections result from impaired mucociliary function [4]; however, in our case, there were no respiratory complications during the perioperative period.

The recognition of anatomical changes and comorbidities by careful preoperative evaluation is necessary to ensure a good clinical course. Anesthetic management of dextrocardia with situs inversus should be performed carefully with various devices. Preoperative ECG with conventional lead placement showed right-axis deviation because of the anatomical mirror position resulting from situs inversus totalis. Therefore, the right ECG lead placement was reversed during and after surgery. Moreover, it should be noted that the positioning of defibrillation pads should also be reversed in patients with situs inversus totalis. Central venous catheterization via the left internal jugular vein has been recommended for patients with situs inversus totalis [4]. We chose the left internal jugular vein for pulmonary artery catheterization to avoid thoracic duct injury. A postoperative chest X-ray showed that the pulmonary artery catheter cannulated through the left internal jugular vein followed a direct course to the superior vena cava, and the tip placement was at the left pulmonary artery.

For surgical procedures in patients with dextrocardia, the presence of the right ventricle on the left side and anterior to the left ventricle, also coursing along the LAD on the right side of the heart, makes revascularization of LAD with LITA difficult because of the short course of the LITA. Usually, the RITA is anastomosed to the LAD, considering its proximity to the rightward LAD [5]. In our case, the CABG anastomoses consisted of RITA-LAD, free LITA-OM, and SVG-RCA, during which the cardiac surgeon stood on the left side of the dextrocardia patient.

Some recommendations regarding the performance of TEE in patients with situs inversus totalis have been reported [6]. TEE views such as the mid-esophageal (ME) 4-chamber view, ME aortic short-axis (SAX) view, and trans-gastric (TG) basal/mid-SAX view appear as mirror images of the normal heart. TEE images obtained at 120° in ME aortic long-axis (LAX) view are obtained at 30–40° in dextrocardia patients. However, TEE views obtained at an angle of 90° in dextrocardia patients are similar to the normal heart [7]. In order to reduce confusion regarding the probe angle adjustment, anesthesiologists may prefer to acquire these views via right-left inversion of the obtained images. Nonetheless, the left-right maneuvering direction of the TEE probe needs to be reversed.

Anesthetic management for cardiac surgery is difficult in patients with situs inversus totalis. However, these patients can be managed more safely with careful preoperative planning and perioperative wise judgment. Our case report describes several important points for ensuring proper anesthetic management of the situs inversus totalis patient during cardiac surgery.

## Conclusion

CABG in dextrocardia with situs inversus totalis can be managed without any complications. Careful perioperative evaluation, intraoperative positioning of the heart, selection of conduits and graft configuration, multiplane angles, and probe adjustments in TEE are needed for these anatomically abnormal patients.

## Data Availability

Please contact the authors for data requests.
